# A Tunable Triple-Band Near-Infrared Metamaterial Absorber Based on Au Nano-Cuboids Array

**DOI:** 10.3390/nano10020207

**Published:** 2020-01-24

**Authors:** Feng Qin, Zeqiang Chen, Xifang Chen, Zao Yi, Weitang Yao, Tao Duan, Pinghui Wu, Hua Yang, Gongfa Li, Yougen Yi

**Affiliations:** 1 Joint Laboratory for Extreme Conditions Matter Properties, Southwest University of Science and Technology, Mianyang 621010, China; qf0510@yeah.net (F.Q.); chenxifang1988@yeah.net (X.C.); myyz1984@csu.edu.cn (T.D.); 2 Research Center for Photonic Technology, Fujian Key Laboratory for Advanced Micro-nano Photonics Technology and Devices & Key Laboratory of Information Functional Material for Fujian Higher Education, Quanzhou Normal University, Fujian 362000, China; czqchem@qztc.edu.cn; 3 State Key Laboratory of Advanced Processing and Recycling of Non-ferrous Metals, Lanzhou University of Technology, Lanzhou 730050, China; hyang@lut.cn; 4 Key Laboratory of Metallurgical Equipment and Control Technology of Ministry of Education, Wuhan University of Science and Technology, Wuhan 430081, China; ligongfa@wust.edu.cn; 5 College of Physics and Electronics, Central South University, Changsha 410083, China; yougenyi@csu.edu.cn

**Keywords:** perfect absorber, surface plasmons, tunable-three band, Au nano-cuboids array

## Abstract

In this article, we present a design for a triple-band tunable metamaterial absorber with an Au nano-cuboids array, and undertake numerical research about its optical properties and local electromagnetic field enhancement. The proposed structure is investigated by the finite-difference time domain (FDTD) method, and we find that it has triple-band tunable perfect absorption peaks in the near infrared band (1000–2500 nm). We investigate some of structure parameters that influence the fields of surface plasmons (SP) resonances of the nano array structure. By adjusting the relevant structural parameters, we can accomplish the regulation of the surface plasmons resonance (SPR) peaks. In addition, the triple-band resonant wavelength of the absorber has good operational angle-polarization-tolerance. We believe that the excellent properties of our designed absorber have promising applications in plasma-enhanced photovoltaic, optical absorption switching and infrared modulator optical communication.

## 1. Introduction

In recent years, there has been a great development of surface plasmons, which is an electromagnetic wave propagating along the interface between a medium and metal. It is generated by coupling the light field and collective electron oscillation on the metal surface [1,2,3,4,5]. Surface plasmons play an extremely important role in optical wave guides, optical absorption switches, subwavelength optics, sensors, and photocatalysis [6,7,8,9,10,11,12,13,14,15]. Local surface plasmon resonance is an important branch of the surface plasmon field. In particular, the local surface plasmon resonance (LSPR) of Au nanoparticles (NPs) has received extensive attention for many years due to its unique optical properties [16,17,18]. Under the action of incident light, the collective oscillation of Au free electrons can enhance the local electromagnetic field on their surface [19]. Au nanoparticles have strong local electromagnetic field enhancement on the surface, which has been widely used in fields such as catalysis, photonics, sensing and surface enhanced Raman scattering (SERS) [20,21,22,23,24,25].

The perfect metamaterial absorbers (MAs) have the ability to absorb incident waves and the property of almost perfect absorption. Moreover, it has some special properties in nature, such as perfect lens, stealth, and negative refractive index [26,27,28], which has attracted wide attention. Metamaterials are composite arrays of subwavelength resonant metal structures with specific optical properties. In 2008, Landy et al. first proposed a perfect absorption material based on a metal structure, which is composed of metal–electrolyte–metal [29]. Due to the strong resonance of metal structures, the absorption rate is almost perfect. The properties of metamaterials depend on the geometry of a single cell. In recent years, people have conducted in-depth studies on the design, preparation and characterization of MAs [30,31,32,33,34,35,36]. Metamaterials have developed from microwave to terahertz, infrared and visible regions [37,38,39,40], and from single band and polarization sensitivity to multiband and weak polarization sensitivity [41,42,43,44]. However, currently existing MAS are mostly single-band, sensitive to polarization and have low tolerance to incident angle, which requires the design of a metamaterial absorber with multiband, insensitive polarization and wide angle.

In recent years, optical absorption studies have been performed on multi-band metamaterial absorbers. For example, Wang et al. explored the absorption rate of triple-band metamaterial [45], but the peak tunability is poor and the sensitivity to angle and polarization is relatively high. In this paper, we address these shortcomings and design a triple-band tunable metamaterial absorber. We adopt the finite-difference time-domain (FDTD) method, and research absorption characteristics and resonance sensitivity to light. We find that the three peaks in the absorption spectrum are caused by resonance effect and surface-based plasmon resonance. The peak can be adjusted by changing the structural parameters of the model and the background environment, and the structure has the advantage of being insensitive to angle and polarization. Therefore, the triple-band tunable ideal metamaterial absorber designed by our team will have potential applications in the fields of plasmon-enhanced photovoltaic, optical absorption switch and infrared modulation optical communication.

## 2. Structure Design and Numerical Model

**Figure 1:**
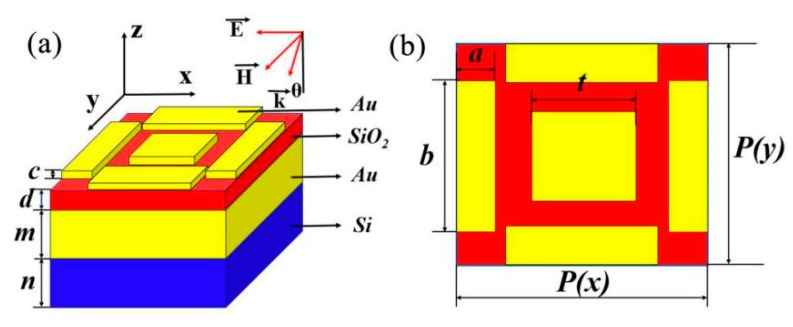
(**a**) Geometric schematic diagram of Au-SiO_2_-Au nano-cuboids array structure. *d* = 35 nm, *c* = 15 nm, m= 200 nm, n = 300 nm. (**b**) Plane diagram of perfect absorber. *P*(*x*) = *P*(*y*) = 600 nm, *t* = 300 nm, *b* = 400 nm, *a* = 90 nm; within the whole structure, Si is the substrate.

In the numerical calculation, Wang et al. used FDTD to calculate the optical properties of the absorber, and we also used the same technique for numerical simulation [46,47]. We numerically analyzed the performance and the optical response of the plasma nanostructures by employing the FDTD simulations. We propose a structure of the tunable triple-band absorber concentrating near the near infrared region as shown in Figure 1. We consider that the metamaterial designed in the top panel contains a square matrix. Gold was chosen as a metallic element for its chemical stability and low ohmic loss. The structure made up of a Au nano-cuboids array. The Drude model is a simulation model that is closer to the experimental item than many other models. For example, Wang et al. used the Drude model to study related absorbers [45]. The dielectric constant of Au can be calculated by using the Drude model [48,49,50], which works well in the near infrared band. The Drude formula is

(1) $$\varepsilon (\omega )=\varepsilon_{\infty }-\frac{\omega_{p}{}^{2}}{\omega^{2}+i\gamma_{c}\omega }$$

where *ω*_p_ is the plasma frequency, and γ_c_ is the damping constant. For Au, *ε_∞_* = 9.1, *γ*_c_ = 4.05 × 10^13^ rad/s, and *ω*_p_ = 1.38 × 10^16^ rad/s [51,52]. The complex refractive index data of Si is taken from Palik [53]. In the metal rectangular plate, *t* represents the length of the side of the rectangular plate with equal sides. *b* and a respectively represent the long side and the short side of the rectangle with unequal sides. During each period, the rectangular plates with equilateral edges are located in the center and the four rectangular plates with unequal edges are located in the four directions of the period. The change in the size of the rectangular plate resonator allows the effective dielectric constant and permeability to be specified. These dielectric constants and permeability provide impedance-matching to minimize reflection. Due to the resonance of the local surface of Au nanoparticles, our absorber can achieve triple-band spectral tuning by adjusting the structure. In the calculation, the plane wave source is polarized along the *x* direction. To reduce computation time and server memory, we consider periodic boundary conditions in the x and y directions. The perfect matching layer can be directly adopted in the z direction outside the structure area. We set the mesh precision to a higher level of 6, and the plane light source used is from 0.9 to 3 µm. The Au nano-cuboids array has the same length along the *x* and *y* directions. The geometric parameters of the structure are set as follows: upper Au thickness *c* = 25 nm, dielectric layer SiO_2_, dielectric layer *d* = 35 nm, lower Au film thickness *m* = 200 nm, substrate SiO_2_ thickness *n* = 300 nm, *t* = 300 nm, *b* = 400 nm, *a* = 90 nm, *P*(*x*) = *P*(*y*) = 600 nm. The absorber we designed is simple in structure, and the preparation process in the experiment is simple and easy to realize.

## 3. Simulations Results and Discussions

**Figure 2:**
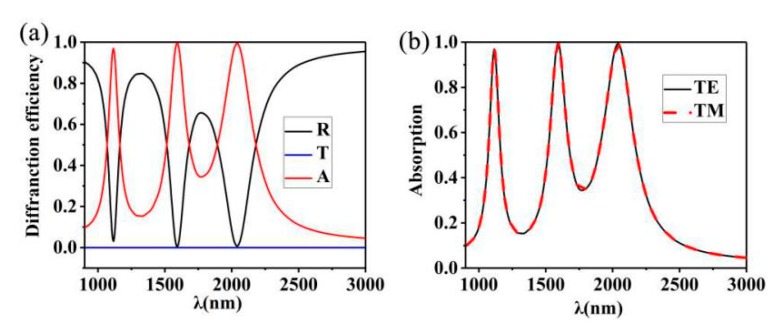
(**a**) Absorption (A), reflective (R), and transmission (T) spectrum of the presented nanostructure. *c* = 25 nm, *d* = 35 nm, *P*(*x*) *= P*(*y*) = 600 nm, *t* = 300 nm, *b* = 400 nm, *a* = 90 nm; (**b**) absorption spectra of the structure in TE and TM modes.

We calculate reflectivity (*R*) and transmittance (*T*) in detail, and then define absorption rate *A* in terms of 1-*T*-*R* [54,55,56,57,58]. The simulation results of the unit units of the proposed nanostructure in the wavelength range of 900–3000 nm are shown in Figure 2a. In the calculation, the plane wave source is polarized along the *x* direction. It can be concluded that in the near infrared range, the transmission quantity is significantly less than 0.01. This is because the thickness of the Au film is greater than the depth of the incident light. The reflection spectrum presents a narrow dip angle at the wavelengths of 1115 nm, 1593 nm and 2039 nm, respectively. The reason for the dip angle of the narrow band is that when the incident light field is incident on the Au nano-cuboids array structure, plasmon resonance will appear in the upper and lower parts of the Au nano-cuboids array, and this plasmon resonance will appear at the three peaks. It can be seen from Figure 2b that since the structure presents periodicity in the direction of TE and TM, this triple-band absorber is insensitive to TE and TM waves.

To evaluate the performance excellence of the proposed structure, we compared the results with other similar absorbers structures with three peaks listed in Table 1. From the table, we can see that two of our peak values are more than 99%, while the others contain at most one. Although our structure is not the highest when comparing the highest peaks, the average peak of the structure we designed is higher than the average absorption peak of other similar structures. It is clear the absorption efficiency of our designed absorber is better than that of the other absorbers in Table 1.

**Figure 3:**
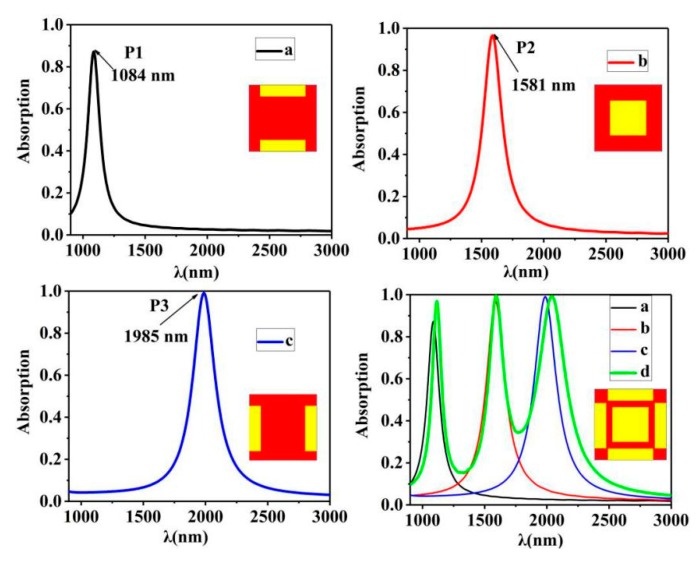
The composition of the structure is (**a**) only non-equilateral rectangular short edge parallel to the *x*-axis of absorption rate; (**b**) only in the middle of equilateral rectangular in the wavelength range of absorption rate; (**c**) only non-equilateral rectangular long edge parallel to the *x*-axis of absorption rate; and (**d**) of the three color dotted line respectively three cases. Figure (**a**–**c**), green solid line is the whole structure in the wavelength range of plasma resonance response.

**Figure 4:**
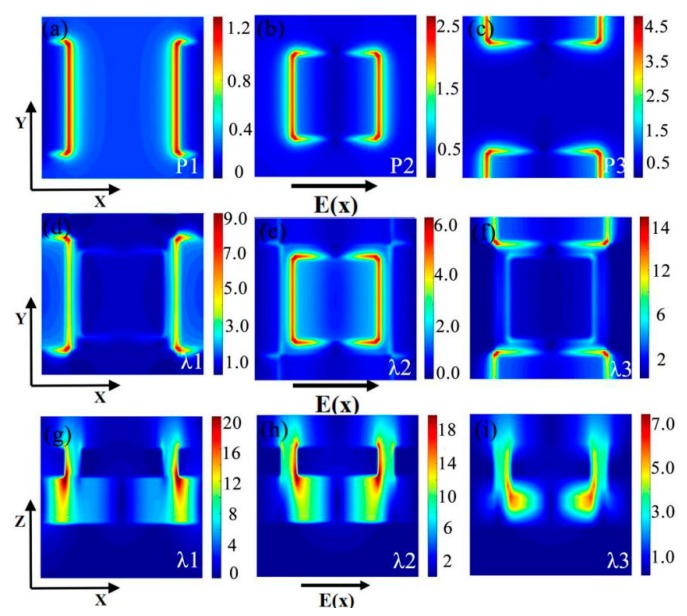
(**a**–**c**) is the electromagnetic field intensity distribution of the array at different shapes of the structure over the period (corresponding to Figure 3a–c) in the *XOY* plane; (**d**–**f**) is the electromagnetic field intensity distribution of the array at different resonant wavelengths (*λ1* = 1115 nm, *λ2* = 1593 nm and *λ3* = 2039 nm) in the *XOY* plane; (**g**–**i**) is the electromagnetic field intensity distribution of the array at different resonant wavelengths (*λ1* = 1115 nm, *λ2* = 1593 nm and *λ3* = 2039 nm) in the *XOZ* plane. The geometric parameters are shown in Figure 2.

In order to better explain the three perfect absorption peaks in the structure we designed previously, we conducted decomposition analysis of the structure. As shown in Figure 3, this is the absorption spectrum diagram of cuboid configurations with different Au nano-particles, and it obtains the response of three bands. According to the simulation results in Figure 3b, when the electric field component of the light source is along the direction of *TE*, the Au nano-cuboids array at the center shows a peak value at 1581 nm. In one period, a peak value was also found on the long side of the marginal cubic structure along the direction of TE, and on the short side of the marginal cubic structure along the direction of TE. It is worth noting that in the simulation, polarized electric field exists along this direction, so we choose to place four rectangular symmetric points. The Au nano-cuboid array is arranged in one period. Meanwhile, we simulate the absorption spectrum of the whole model by maintaining the incident polarization. As can be seen from Figure 3d, the green line is compared with the lines of other colors: the three peaks all show a slight blue shift; it is worth noting that the green line has a much higher peak than the black line (from 0.87 to 0.97), this is because the structure of the surface plasmon resonance peak and part of the plasma enhanced effect appears. Since the three peaks all correspond to each other, we can determine that the absorption spectra of the three bands are composed by Figure 4a–c.

In addition, in order to better understand the physical mechanism of peaks, we calculate the Figure 3a–c the corresponding electric diagram in the *XOY* plane. In Figure 4a–c, by comparing the two figures find that when the peak at 1084 nm, a peak perpendicular to the long side of the *X*-axis cube resonance formed. When the peak is at 1581 nm, the peak is formed by the resonance of the middle cube. The other dipole plasma resonance was found to be generated by the short side of the cube perpendicular to the *X*-axis at 1985 nm. At the same time, we also calculate the electromagnetic field intensity distribution at different resonant wavelengths (*λ1* = 1115 nm, *λ2* = 1593 nm, *λ3* = 2039 nm) in the *XOY* plane (Figure 4d–f). As shown in Figure 4e, the peak at 1115 nm by complete perpendicular to the x-axis of the cube long edge to the formation of resonance. Similarly, the peak at 1593 nm is basic caused by the resonance of the intermediate cube, the peak at 2039 nm is basic caused by the short side resonance of the cube perpendicular to the x-axis; this also further confirmed that the peak value of the three sources are respectively formed by the separate cube resonance. It is noteworthy that the resonance effect of Figure 4e–f is stronger than that of Figure 4a–c, which is caused by the interaction between the cube resonance during this period. Meanwhile, we calculate the electromagnetic field intensity distribution at different resonant wavelengths (*λ1* = 1115 nm, *λ2* = 1593 nm, *λ3* = 2039 nm) in the XOZ plane. It can be seen from Figure 4g–i that the strong electromagnetic field is mainly concentrated between the Au/SiO_2_ interface and the air/metal nano-cube at the top. Under the action of incident plane waves, it can be seen from Figure 4g that the strong electromagnetic field is excited by the Au cube and SiO_2_ on both sides, while in Figure 4h, the excitation is mainly caused by the interaction between the Au cube, SiO_2_ and air. For *λ3*, it is not difficult to see the interaction between the Au cube and SiO_2_ under the action of incident waves through Figure 4i. From Figure 4, it can be concluded that the surface plasmon polaritons’ (SPPs) resonance and Fabry-Pero resonance show that there will be a strong electromagnetic field distribution near the nano scale groove [61,62,63].

**Figure 5:**
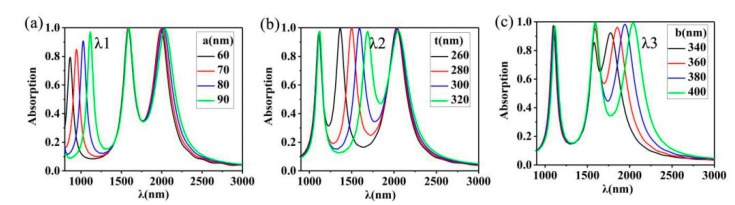
(**a**) The linear relationship between the absorption spectral deviation of different *a* values. (**b**) Spectral shift of three modes for different values of *t*. (**c**) The linear relationship between the absorption spectral deviation of different *b* values.

We also explored the effects of different parameters on the absorption of the Au nano-cuboids array, as shown in Figure 5b; *t* = 260 nm (black), *t* = 280 nm (red), *t* = 300 nm (blue), *t* = 320 nm (green). Due to the close proximity of the Au nano-cuboids array, the coupling effect may affect our absorption performance. Therefore, we selected the changes of fixed geometric parameters that will affect resonance. First, we fixed the parameters of the surrounding cube within the period, *a* = 90 nm and *b* = 40 nm, and changed the side length of the middle cube in the period, as shown in Figure 5b. As *t* increases from 260 to 320 nm, the corresponding resonance peak showed obvious red shift (*λ2*: from 1358 to 1658 nm). Then, as shown in Figure 5a, by changing the value of side length *a*, we can clearly see that *λ1* varies from (863, 0.78), (943, 0.84), (1027, 0.90) to (1111, 0.96). The peak value shows obvious red shift and rise and presents a linear relationship. As shown in Figure 5c, by changing the value of side length *b*, we can see that *λ3* changes from (1772, 0.92), (1584, 0.96), (1940, 0.98) to (2039, 0.99), and the peak value shows obvious red shift and rise. It can only be noted that there is a certain increase in the peak value of *λ2* here (from 0.85 to 0.99), which is due to resonance. These indicate that we can adjust the position and height of the three peaks by changing the structure so that we can use them as tunable metal elements such as optical absorption switches and optical modulators [64,65,66].

**Figure 6:**
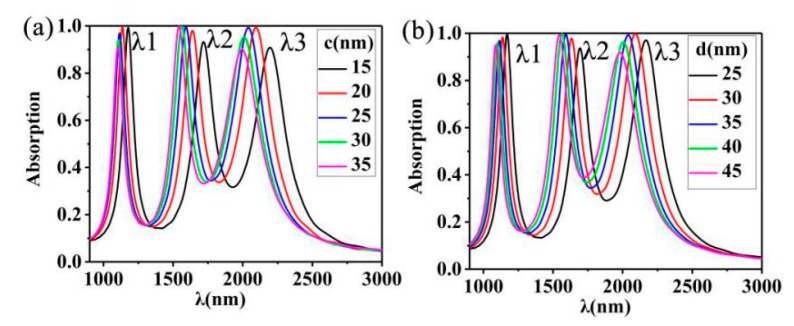
(**a**) The simulated absorption spectrum of the metamaterial absorber changes with the thickness of the top layer Au film *c* is 15, 20, 25, 30, and 35 nm, and other geometric parameters are the same as those in Figure 2b. (**b**) The absorption spectrum changes with the thickness of the dielectric layer SiO_2_ *d* is 25, 30, 35, 40, and 45 nm, with other parameters unchanged.

The effects of the thickness of the dielectric layer and the thickness of the Au nano-cuboids array on the absorption spectrum were also studied. The results are as shown in Figure 6, through preliminary observation, we can find that changing the thickness of dielectric layer *c* and changing the thickness of top layer *d* have very similar effects on our absorption rate. There is a slight blue shift and a decrease in the peak value of *λ1*, *λ1* in Figure 6a from (1185, 0.99) to (1079, 0.89), and *λ1* in Figure 6b from (1167, 0.99) to (1084, 0.94). For *λ2*, there is a blue shift and a decrease in the peak value of *λ2*. *λ2* in Figure 6a from (1716, 0.93) to (1540, 0.99), and *λ2* in Figure 6b from (1697, 0.93) to (1542, 0.99). For *λ3*, blue shift occurs. It is worth noting that the peak value of *λ3* increases first and then decreases as the value of c or d changes. With the characteristics of the medium layer and top metal, we can also make tunable metal elements.

**Figure 7:**
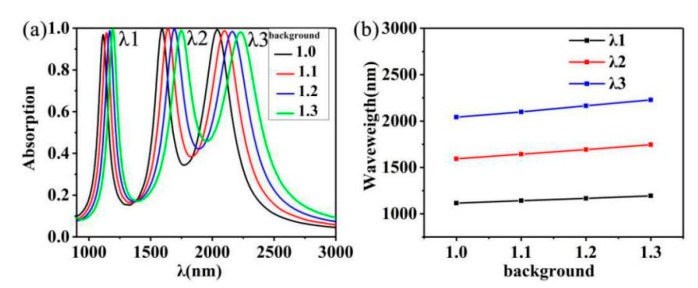
(**a**) Different absorption spectra of different background environments (1.0–1.3). (**b**) A linear relationship of resonance wavelengths in response to changes in background environments.

However, for many metal absorbers, once their structures are fixed, their resonant wavelengths will no longer be tunable, so we calculated the change of absorption spectrum in different background environments. Figure 7a shows the absorption spectra of *n* = 1.0, *n* = 1.1, *n* = 1.2, and *n* = 1.3 with wavelengths of 900–3000 nm. The phenomenon of red shift appears with the increase of the ambient refractive index by three peaks. *λ*1 from 1115 nm to 1190 nm, *λ2* from 1593 to 1743 nm, *λ3* from 2093 to 2224 nm. It can also be seen from Figure 7b that the redshifts of the three absorption peaks vary linearly from 1.0 to 1.3 in the background environment. Therefore, this resonance mode of our designed absorber can be used to adjust our absorber to achieve the desired effect in different environments.

**Figure 8:**
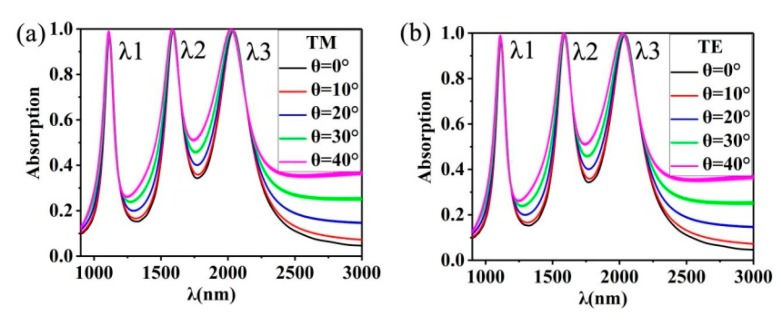
(**a**) Absorption spectra of the structure designed for us at 0°~ 40° in TM wave; (**b**) Absorption spectra of the structure designed for us at 0°~ 40° in TE wave.

It is well known that in practical applications it is very important for devices to be insensitive to angle and polarization [67,68,69]. Therefore, we performed additional simulation calculations to study the effects of angle and polarization on triple-band absorbent materials. It can be seen from Figure 8a,b that when the incidence angle reaches 40°, the perfect absorption is almost uninterrupted while keeping the central wavelength unchanged. This means that these strong absorbents are weak depending on polarization and incidence angle. This is because the Au nano-cuboids array we designed has good symmetry and can be completely absorbed by the absorber. The above results show that the triple-band tunable ideal absorber with nanostructured Au has a good angular polarization tolerance, which overcomes one of the shortcomings of the former absorber.

## 4. Conclusions

In conclusion, we designed a triple-band metamaterial absorber which is weakly dependent on polarization and incident angle. It consists of three layers of thin films called Au–SiO_2_–Au. Through the numerical calculation of the absorber, the results show that due to the Fabry–Pero resonance effect, the strong field is localized in SiO_2_ in the dielectric layer. The SiO_2_ layer limits the action of the electromagnetic field. The results show that the three absorption peaks of 96.8%, 99.6% and 99.2% can be achieved by adjusting the Au nano-cuboids array and film thickness. In addition, our absorber has the advantage of a good angular polarization tolerance. Through the reasonable design of the geometric structure of the device, the device can be used in the near-infrared resonance absorption wavelength. The results show that the periodic tunable triple-band metamaterial absorber has the advantages of perfect absorption, insensitivity to angle and polarization, and it can be widely used in plasma enhancement photoelectric, light absorption switch, infrared modulation light communication and other fields.

## Figures and Tables

**Table 1:** Comparison results of presented absorber with other similar triple-band absorbers [45,59,60].

Refer	Number of Peaks	The Value of the First Peak	The Value of the Second Peak	The Value of the Third Peak
[45]	3	96.4%	96.3%	96.7%
[59]	3	97.6%	96.5%	84.1%
[60]	3	96.9%	94.3%	99.8%
Present	3	96.8%	99.6%	99.2%
